# Is intraoperative parathyroid monitoring during minimally invasive parathyroidectomy still justified?

**DOI:** 10.3389/fendo.2024.1442972

**Published:** 2024-07-22

**Authors:** Lindsay Hargitai, Daniela Boryshchuk, Melisa Arikan, Teresa Binter, Christian Scheuba, Philipp Riss

**Affiliations:** ^1^ Department of General Surgery, Division of Visceral Surgery, Medical University Vienna, Vienna, Austria; ^2^ Center for Medical Data Science, Institute of Medical Statistics, Medical University Vienna, Vienna, Austria

**Keywords:** primary hyperparathyroidism, minimal invasive parathyroidectomy, intraoperative parathyroid hormone assay, parathyroid adenoma, preoperative localization studies, ultrasound, MIBI, PET-CT

## Abstract

**Introduction:**

Primary hyperparathyroidism (PHPT) is the third most common endocrine disease. With parathyroidectomy, a cure rate of over 95% at initial surgery is reported. Localization of the abnormal parathyroid gland is critical for the operation to be successful. The aim of this study is to analyze data of patients with single gland disease (SGD) and positive concordant localization imaging undergoing minimally invasive parathyroidectomy (MIP) and intraoperative parathyroid hormone monitoring (IOPTH) to evaluate if IOPTH is still justified in patients with localized SGD.

**Methods:**

A retrospective database analysis of all minimally invasive operations with IOPTH for PHPT and positive concordant localization in ultrasound (US) and ^99m^Tc-sestamibi scintigraphy (MIBI) between 2016-2021. When both US and MIBI were negative, patients underwent either choline or methionine PET-CT. The patients were also analyzed a second time without applying IOPTH.

**Results:**

In total, 198 patients were included in the study. The sensitivity of US, MIBI and PET-CT was 96%, 94% and 100%, respectively. Positive predictive value was 88%, 89% and 94% with US, MIBI and PET-CT, respectively. IOPTH was true positive in 185 (93.4%) patients. In 13 (6.6%) patients, no adequate IOPTH decline was observed after localizing and extirpating the assumed enlarged parathyroid gland. Without IOPTH, the cure rate decreased from 195 (98.5%) to 182 (92%) patients and the rate of persisting disease increased from 2 (1.0%) to 15 (7.5%) patients.

**Conclusion:**

Discontinuing IOPTH significantly increases the persistence rate by a factor of 7.5 in patients with concordantly localized adenoma. Therefore, IOPTH appears to remain necessary even for this group of patients.

## Introduction

Primary hyperparathyroidism (PHPT) is the third most common endocrine disease. A cure rate of over 95% at initial surgery can be achieved with parathyroidectomy (PTx) (1).

Localization of the abnormal parathyroid gland is crucial for the operation to be successful. However, this can be difficult due to their unpredictable location following migration in the embryonic period. Anomalies in parathyroid numbers are observed in 3–6% of individuals (2). Intraoperative monitoring of parathyroid hormone (IOPTH) during parathyroidectomy serves as an adjunctive method to confirm a successful cure or detect any remaining hyperfunctional parathyroid tissue after the removal of the diseased gland. With the introduction of IOPTH, patients with successful preoperative localization of the parathyroid adenoma can undergo minimally invasive parathyroidectomy (MIP). Studies have demonstrated that IOPTH enhances the effectiveness of MIP by increasing the cure rate and reducing the likelihood of reoperation (3, 4). The advantages of MIP over classical open PTx include reduced operation time, fewer complications (1-3%), reduced costs, and patients can be operated on as an outpatient basis (5). Furthermore, MIP has a high success rate of 95-98%, similar to classic PTx (5). A classic open PTx is conducted if localization diagnostics are negative and if hyperplasia or familial disease is suspected or has been diagnosed (5).

The most employed preoperative localization imaging studies include neck ultrasound and ^99m^Tc-sestamibi scintigraphy. The overall accuracy of ultrasound is 88%, with a range between 76-87% and positive predictive value (PPV) of 93-97% (6). ^99m^Tc-sestamibi scintigraphy has a high accuracy of 97%, with a high sensitivity of 90% (7). A combination of ultrasound and sestamibi produce the highest sensitivity over just one diagnostic procedure (8, 9). With technology advancing, the resolution of both ultrasound and MIBI are progressing, which in turn can improve their accuracy in identifying enlarged parathyroid glands. When both MIBI and US are negative, patients receive either ^11^C-Methionine PET/CT scintigraphy or ^18^F-Cholin PET-CT. PET-CT has numerous advantages over conventional preoperative imaging including shorter execution times, higher efficiency and lower radiation dose with higher resolution imaging (10–12). Furthermore, PET-CT is the best preoperative imaging modality for identifying ectopic adenomas and less operator dependent than US (11, 13). The results of previous studies reinforce the awareness of PET-CT as a first-line preoperative imaging option (11, 14).

However, even the application of both ultrasound and sestamibi and PET-CT cannot totally exclude multiple gland disease (MGD) (8, 9). Therefore, the application of IOPTH monitoring during the operation is recommended to rule out MGD even in patients with presumed single gland disease (SGD) (15, 16). Given that technology and diagnostic imaging is constantly advancing, and similar long-term results have been described in the literature with (17) and without (18–20) IOPTH, it might be questioned, whether IOPTH is really essential in localized SGD. According to the German Association of Endocrine Surgeons (CAEK) guidelines, surgeons can choose not to employ IOPTH in patients with concordant localization in SGD (21), however, a previous published study demonstrated that IOPTH still needs to be employed in patients with concordant localized SGD (22). On the other hand, the American Association of Endocrine Surgeons recommend using IOPTH when planning an image-guided focused parathyroidectomy in order to prevent higher rates of operative failure (23). This study analyzed all PHPT patients undergoing MIP and IOPTH who underwent sestamibi and ultrasound or PET-CT on newer devices than in a previous study (22) with the aim to evaluate if IOPTH monitoring is truly justified in patients with concordantly localized SGD.

## Materials and methods

### Patients

All consecutive patients between 2016-2021 with the biochemical diagnosis of PHPT who underwent parathyroid surgery at a tertiary referral care center (university hospital) were included in this single center analysis. For this analysis, only patients with a follow-up of at least two years were included. All patients had localized single gland disease (concordant MIBI/US and/or positive localization in PET/CT) were scheduled to undergo MIP: OMIP (open minimally invasive parathyroid exploration)/UNE (unilateral neck exploration) with the use of IOPTH monitoring. According to national (21) and international (23, 24) guidelines, all patients met the criteria for PTx. Exclusion criteria were patients with hereditary disease (MEN, FHH, familiar HPT), parathyroid carcinoma, ectopic adenoma in thorax, no preoperative imaging or discordant MIBI/US results and suspected MGD or bilateral thyroid nodules requiring initial bilateral neck exploration (BNE). In case of thyroid disease documented by ultrasound (US) with an indication for thyroid surgery, an ipsilateral hemithyroidectomy (lobectomy with isthmus) was performed and were included in the study after chart review. All patients were treated by the same diagnostic and surgical protocol.

### Preoperative localization and surgical strategy

All patients preoperatively underwent a standard double phase Tc-99mhexakis-2-methoxyisobutylisonitrile scintigraphy (99mTc-sestamibi-scan) with SPECT (MIBI) and high-resolution US with color Doppler of the cervical region to localize one or more enlarged (presumed hyperfunctioning) parathyroid gland(s) and to evaluate the morphology of the thyroid gland [see standard protocols (15, 25)]. US examinations were performed by an experienced radiologist, MIBI by an independent nuclear medical physician. If both US and MIBI results were negative, patients underwent a PET-CT scan using either ^11^C-Methionine or ^18^F-Choline as tracers. All test results were discussed with the surgeon the day before surgery.

Patients with localized SGD on MIBI and US or PET-CT underwent MIP (OMIP/UNE). Only the localized enlarged parathyroid gland was identified and extirpated, and the operation was then completed. If there was no appropriate decline in IOPTH indicating MGD, MIP was converted to a BNE. In patients with additional thyroid surgery, a hemithyroidectomy was performed after removing the enlarged parathyroid gland and after interpretation of IOPTH.

### IOPTH monitoring

A commercially available two-step sandwich PTH STAT ECLIA (electrochemiluminescence immunoassay) (Roche^®^ Diagnostics Mannheim, Germany) run on the Cobras e411 analyzer was used in all patients to determine preoperative, intraoperative and postoperative PTH. The intra-assay coefficient of variation is =<1.8% and the inter-assay coefficient of variation is =<2.5%.

Peripheral blood samples were taken at the beginning of the operation (baseline), at the time of extirpation of the enlarged gland(s) and 5, 10 and 15 minutes after extirpation. Further blood samples were taken in patients with intraoperative “PTH spikes” (26).

### Definition of cure

A decay of ≥50% from the “baseline value” within 10 minutes after extirpation defined the complete resection of the parathyroid tumor and therefore cure (“Vienna criterion”) (15).

### Follow-up

Both Calcium (Ca) and parathyroid hormone (PTH) were measured in all patients on post-operative days 1, 4, 7, 6 weeks, and 6 and 12 months, respectively after surgery and then only once a year.

“Cured” patients were defined as those whose Ca and PTH values were within the normal range during follow-up. “Persisting disease” was defined as Ca and PTH still elevated at 6 months following surgery. Patients in whom Ca and PTH values were initially documented in the normal range for at least 6 months and in whom an increase in Ca and PTH levels was later observed were defined as “disease recurrence”. Permanent hypocalcemic patients presented with Ca and PTH below normal range as well as hypocalcemic symptoms necessitating Ca and vitamin D3 supplementation to maintain Ca in the normal range at 12-month follow-up.

Mean follow-up was 11.3 ± 6.2 months (range 1-43 months).

### Statistics

In an initial analysis, patients were evaluated for rate of permanent normocalcemia and conversion rate after applying IOPTH monitoring (IOPTH group). In a second analysis, patients were re-evaluated as if no IOPTH monitoring had been used (non-IOPTH group). McNemar’s test was used to compare the proportions of patients with normocalcemia in the two paired groups (with IOPTH and without). A p-value of less than 0.05 was considered statistically significant.

### Descriptive statistics

The characteristics of the study population were described as following: categorical variables were summarized using frequency tables and percentages, while continuous were summarized using median, first and third quartiles.

## Results

### Patients

**Figure 1:**
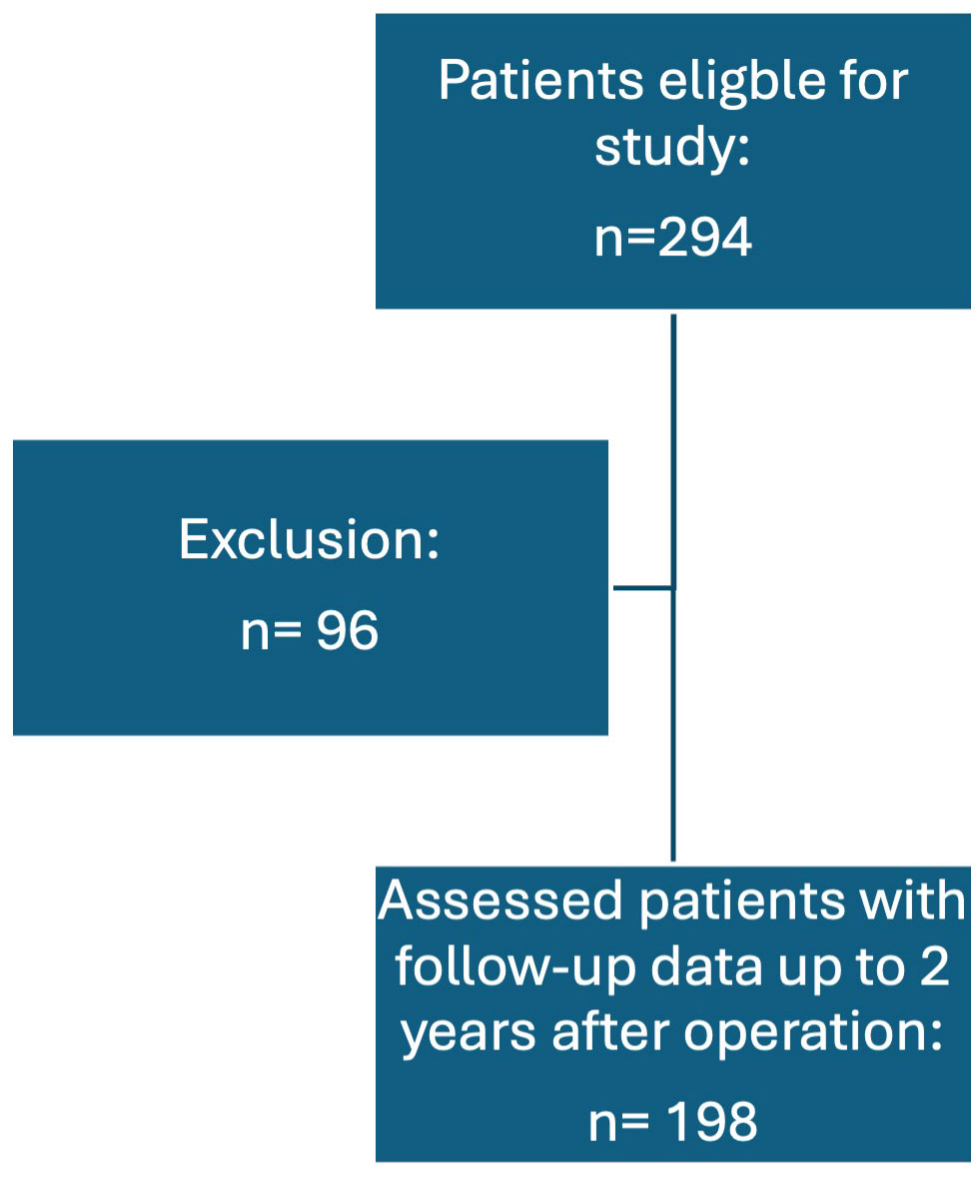
Flow-chart of patient selection.

During the study period, a total of 294 primary operations for PHPT were performed. Of these patients, 198 (67.3%) had concordant localization studies (localized SGD) or positive localization in PET-CT and were therefore included. Ninety-six (32.7%) had either a negative localization, no concordant localization studies or underwent a conventional BNE due to bilateral thyroid disease, thus they were excluded from this study, see **Figure 1**. In total, 149 (75.2%) of the patients were female and 49 (24.8%) were male. The most common symptom reported was osteopenia/osteoporosis in 55 (31.6%) patients, followed by no symptoms in 41 (23.6%) and lastly fatigue in 22 (19%) patients.

### Preoperative imaging

In this analysis, US and MIBI were conducted in 193 (97.5%) and 195 (98.5%) patients, respectively. US exhibited a true positive (TP) location in 165 (83.3%) of patients, a false positive (FP) location in 23 (11.6%) patients and was false negative (FN) in seven (3.6%) patients. Missing data on preoperative localization in three (1.5%) patients was observed. Thus, the overall sensitivity of US was 96% and positive predictive value (PPV) was 88%. In terms of preoperative MIBI, a TP location was observed in 162 (81.8%) of patients, FP in 20 (10.1%) patients and FN in 11 (5.6%) patients. Missing data was seen in five (2.5%) patients. Therefore, the overall sensitivity and PPV of MIBI was 94% and 89%, respectively. When both preoperative imaging modalities were negative, patients underwent either ^11^C-Methionine or ^18^F-Choline PET-CT. In total, 17 (8.6%) PET-CT preoperative examinations were conducted, 16 of which exhibited TP locations of the enlarged parathyroid gland, one PET-CT was determined to be FP. Thus, the overall sensitivity of PET-CT was 100% and PPV was 94%.

### Surgery

In total, MIP (OMIP or UNE) was conducted in 180 (90.9%) patients, and in 18 (9.1%) patients, a conversion to BNE was required. OMIP was planned in 119 (60.1%) patients and UNE in 61 (30.8%) patients. Additional ipsilateral thyroid surgery due to thyroid nodules diagnosed preoperatively via US was carried out in 38 (19.2%) patients. In total, 13 (6.6%) patients had false-positive intraoperative localizations, only two (15.4%) of which demonstrated additional thyroid pathologies. Of these 13 patients, two (15.4%) underwent a conversion a UNE and 10 (77%) converted from OMIP/UNE to BNE. Of the 185 patients with correct localization using IOPTH, 177 (95.7%) underwent planned OMIP/UNE, six (3.2%) patients required a conversion from OMIP to BNE and two (1.1%) patients a conversion from UNE to BNE. Thus, 8 (4.3%) patients with concordant positive localization and IOPTH required a more extended surgical procedure than originally planned. This conversion rate was not statistically significant (p=0.0639). Thyroid pathologies were observed in 36 (19.5%) of 185 patients in which IOPTH was also true positive. There was no significant difference between the two groups (IOPTH/no IOPTH) and the presence of thyroid pathologies. Thirty-three (91.7%) of these patients underwent planned OMIP/UNE, and only three (8.3%) patients a conversion from OMIP/UNE to BNE.

### MIP with IOPTH monitoring (IOPTH group)

IOPTH correctly identified the enlarged single adenoma at the predicted localization in 185 (93.4%) of 198 patients. In 176 (88.9%) patients, the standard interpretation of IOPTH decay determined a complete resection of all hyperfunctioning tissue. In 13 (6.6%) of 198 patients, no adequate IOPTH decline was observed indicating persisting disease due to multi-gland disease (MGD). A conversion of the initial operation technique was conducted in all 13 patients where double adenomas were found in nine (4.5%; of 198 patients) patients and MGD in four (2.0%; of 198 patients) patients. The second enlarged parathyroid gland was found on the contralateral side and impacted in the thyroid gland in two patients, in a contralateral upper deep paraoesophageal position in one patient, on the contralateral lower side in one patient and on the ipsilateral side in five patients. At 6 months follow-up, all of these patients were normocalcemic without any form of calcium or vitamin D therapy and were all classified as cured.

In the three of the four patients with MGD, a subtotal parathyroidectomy (removal of 3.5 parathyroid glands) was conducted. At 6 months follow-up, all three patients were normocalcemic and classified as cured. In one of the four patients, only three enlarged glands were extirpated. At 6 months follow-up, this patient was also normocalcemic and therefore also considered cured.

Two (15.4%) of 13 patients with false-positive localizations had additional thyroid lesions, while thyroid abnormalities requiring ipsilateral thyroid surgery in patients with true positive intraoperative parathyroid localization with IOPTH were observed in 36 (19.5%) patients.

Preoperative Ca and PTH levels of the patients with false-positive localization did not significantly differ from patients with correct localization.

In terms of follow-up in the IOPTH group, an overall cure rate was observed in 195 (98.5%) of patients. Hypercalcemia and elevated PTH levels were observed in two (1.0%) patients. In one of these patients the operation technique was converted from OMIP to BNE and one gland was removed. Preoperative localization with MIBI and US were both false positive in this patient. The second patient in which persistence was observed underwent a planned UNE where one gland was removed. In this patient preoperative imaging with MIBI and US were also false positive. In both patients, IOPTH demonstrated adequate decline according to the Vienna criterion. Within the whole group, only one patient (0.5%) suffered a recurrence. By definition, this recurrence was observed six months after surgery. This patient underwent OMIP with removal of one gland. Both preoperative imaging with MIBI and US, as well as IOPTH were true positive. Out of the entire patient population 189 (95.5%) of 198 patents had parathyroid adenomas in the final histology, the remaining 9 (4.5%) parathyroid hyperplasia.

### MIP without IOPTH monitoring (non-IOPTH group)

In 13 (6.6%) of 198 patients, no adequate intraoperative PTH decline was observed after localizing and extirpating the assumed enlarged parathyroid gland. Therefore, the cure rate decreased to 182 (92%) and the rate of persisting disease increased to 15 (7.5%) patients. The number of patients with recurrent disease would still be one (0.6%) patient.

When analyzing the results of the IOPTH group versus non-IOPTH group, the number of patients with persisting disease increased significantly from 1.0% (2/198) to 7.5% (15/198) (p = 0.000874; McNemar’s test), see **Table 1**.

**Table 1:** IOPTH group versus non-IOPTH group: long-term follow-up results.

Follow-up^a^	IOPTH group	Non-IOPTH group	p^b^
Cure rate	195 (98.5%)	182 (92.0%)	0.0008741
Persistence	2 (1.0%)	15 (7.5%)	0.0008741
Recurrence	1 (0.5%)	1 (0.5%)	n.s.^c^
Sum	198 (100%)	198 (100%)	n.s.

a Mean follow-up: 9.7 ± 11.5 (range 1-126) months.

b McNemar’s test, p<0.05.

c n.s. not significant.

When analyzing the final histology of the patients without IOPTH monitoring, 9 (69.2%) had parathyroid adenomas and 4 (30.8%) parathyroid hyperplasia.

## Discussion

In this study, 198 (67.3%) of 294 patients with concordant localization MIBI and US or positive localization in PET-CT underwent primary operation for PHPT in an endemic goiter region. In total, 149 (75.2%) of the patients were female and 49 (24.8%) were male. In this analysis, the majority of patients suffering from PHPT are female and over the age of 60 years, in line with the literature, where PHPT is typically diagnosed in postmenopausal women (27).

Preoperative imaging is employed to localize the presumed enlarged gland and thus limit the surgical field for the surgeon. In 165 patients (83.3%), US correctly identified the presence of the enlarged gland (TP), while in 23 patients (11.6%) it incorrectly indicated its presence (FP), and in seven patients (3.6%) it missed detecting it (FN). Additionally, three patients (1.5%) had missing preoperative localization data. Consequently, US demonstrated an overall sensitivity of 96% and a positive predictive value (PPV) of 88%, both of which lie within the literature rates (28–30). Although US offers advantages of affordability, non-invasiveness, and rapidity, its sensitivity may diminish in instances of multiple parathyroid adenomas, parathyroid hyperplasia, or concurrent thyroid nodules (31, 32). Moreover, its efficacy is contingent upon the operator, and distinguishing between parathyroid adenomas and lymph nodes can pose challenges (33). Preoperative MIBI imaging showed a TP location in 162 patients (81.8%), FP in 20 patients (10.1%), and FN in 11 patients (5.6%), with five patients (2.5%) having missing data. Thus, MIBI exhibited an overall sensitivity of 94% and a PPV of 89%, both of which fall within previously reported ranges (29, 30). Research indicates that thyroid adenomas, thyroid nodules, and thyroid cancer can lead to elevated uptake without subsequent washout, resulting in false positive and potentially false negative outcomes (34). However, a recent study found that the presence of concurrent thyroid nodules in patients with PHPT, whether benign or malignant, did not impact the sensitivity of MIBI (35). False negative results may however occur due to small parathyroid adenomas and ectopic adenomas (36). A notable drawback of both US and MIBI is their incapacity to detect patients with MGD (8). Thus, in this study, when both preoperative imaging modalities yielded negative results, patients underwent either ^11^C-Methionine or ^18^F-Choline PET-CT. Of the 17 PET-CT preoperative examinations conducted (8.6% of cases), 16 correctly identified the enlarged parathyroid gland (TP), while one was deemed a FP. This resulted in the highest sensitivity of 100% and PPV of 94%, both aligning with previous studies (10, 30). Although not the primary imaging modality for patients with PHPT, PET-CT offers several advantages over traditional preoperative imaging methods, including quicker execution, enhanced efficiency, diagnosis of MGD and lower radiation exposure coupled with superior resolution imaging (10–12, 37, 38). Additionally, PET-CT excels as the optimal imaging technique for detecting ectopic adenomas and is less reliant on operator proficiency compared to US (11, 13). Despite its associated higher costs and limited availability, it is crucial to recognize that the initial investment in PET-CT imaging may correlate with increased rates of preoperative parathyroid localization, thereby potentially reducing overall treatment expenses.

Thyroid abnormalities were detected in 36 (19.5%) out of 185 patients with successful localization of the enlarged parathyroid gland using IOPTH. No significant difference was found between the MIP and conversion group regarding thyroid pathologies. However, given that there were only two patients in the conversion group with thyroid nodules, the lack of statistical significance needs to be analyzed in further studies with larger patient populations.

While there is no “one criterion fits all” when it comes to IOPTH monitoring for hyperparathyroidism, several studies have exhibited the increased value of employing IOPTH in order to prevent reoperation in 3-9.6% of patients (17, 39). The overall success rate of IOPTH for spontaneous PHPT lies between 97-99% (40–43), while the failure rate of selective parathyroidectomy (SP) without IOPTH lies between 1-2.7% (18, 20). Although a previous published study by Riss et al. (22) demonstrated that IOPTH still needs to be employed in patients with concordant localized SGD, other studies have questioned if IOPTH is truly required in patients with concordantly localized SGD, given that it can increase costs, operative time and conversion rate due to inadequate intraoperative decline (17, 18, 20, 29, 44). Even international guidelines have started to leave the decision of the utilization of IOPTH with the surgeon (21).

All patients met the criteria for PTx as outlined by national (21) and international (23, 24) guidelines. As per the ESES consensus statement, if the abnormal parathyroid gland(s) can be identified pre-operatively, a focused approach is advised (45). In this study, out of 185 patients with accurate localization using IOPTH, 177 (95.7%) underwent planned OMIP/UNE, whereas 8 (4.3%) patients with positive localization and IOPTH required a more extensive surgical procedure than initially planned. This conversion rate did not reach statistical significance (p=0.0639), which has been also observed in previous studies (22).

In the whole cohort, 195 (98.5%) patients were cured and three (1.5%) demonstrated persistence or recurrence. The overall cure rate was very high and lies within the literature and a recently published meta-analysis (4, 46). Interestingly, the cure rate in this study is higher than was observed in a previous study (22). This may be due to the fact that patients in this study not only underwent preoperative localization studies with the newest US and MIBI equipment; patients with negative localization received PET-CT, which identified the enlarged parathyroid glands in 16 (94.1%) of 17 patients. In the study by Riss et al. (22) PET-CT was not employed in patients with negative localization in US and MIBI.

In total, 13 (6.6%) patients did not exhibit a sufficient decline in intraoperative PTH levels following localization and removal of the suspected enlarged parathyroid gland. Thus, the cure rate decreased to 182 patients (92%), with 15 patients (7.5%) experiencing persistent disease. The number of patients with recurrent disease remained at one (0.6%). In this study and in accordance to previous studies (4, 22, 47), the benefit of IOPTH in patients with localized SGD was exhibited, as the rate of persisting disease would significantly increase from 1.0% (2/198) to 7.5% (15/198) (p= 0.000874). Thus, the overall cure rate would decrease from 98.5% to 92%. These results are similar to recently published studies by Akgün et al. (48) and Quinn et al. (4), which demonstrated an increase in cure rate by 4.3% and 3.2%, respectively, when IOPTH was employed in patients with SGD and concordant localization. Furthermore, the results of this study confirm the efficacy of IOPTH in decreasing the incidence of persistence disease, as was also observed in a meta-analysis by Medas et al. (3). As seen in the meta-analysis by Quinn et al. (4), the higher persistence rate observed in patients that did not receive IOPTH was in turn associated with a higher re-operation rate, which results in greater hospital costs and postoperative morbidity. While studies have suggested that the use of IOPTH itself results in higher costs due to longer operation times, Quinn et al. (4) determined that the difference in mean operation time between the use of IOPTH and no IOPTH was not significant. Given that the patients in the IOPTH group were less likely to undergo re-operation, costs associated with a further procedure and possible postoperative morbidity, as well as the management of persistent hypercalcemia are therefore circumvented (4).

The primary limitations of this study include its single-center design and retrospective nature. Although all parameters were collected prospectively, the interpretation of the results were performed retrospectively and thus did not influence the surgical strategy of the patients. Nonetheless, the importance of IOPTH to the surgical success in patients with localized SGD and positive preoperative localization was examined this study.

This study advises the continued use of IOPTH in SGD patients with localized disease, in accordance with the recommendation of the American Association of Endocrine Surgeons (23) and the proposal by Medas et al. (3) to suggest and implement the routine use of IOPTH in surgery for PHPT.

## Conclusion

IOPTH in patients with localized SGD should further be employed. By discontinuing IOPTH, the persistence rate can significantly increase by a factor of 7.5. Although there isn’t a universal criterion suitable for all cases of IOPTH monitoring in patients with primary hyperparathyroidism, numerous studies, including this one, have highlighted the enhanced efficacy of utilizing IOPTH to minimize the need for reoperation.

## Data availability statement

The raw data supporting the conclusions of this article will be made available by the authors, without undue reservation.

## Ethics statement

The studies involving humans were approved by Ethics Committee of the Medical University of Vienna. The studies were conducted in accordance with the local legislation and institutional requirements. Written informed consent to all diagnostic and surgical procedures as well as possible use of their data for future publications was collected from all patients.

## Author contributions

LH: Conceptualization, Data curation, Investigation, Methodology, Writing – original draft, Writing – review & editing. DB: Formal Analysis, Writing – review & editing. MA: Writing – review & editing. TB: Writing – review & editing. CS: Supervision, Writing – review & editing. PR: Supervision, Writing – review & editing.
